# Lumpy skin disease virus LSDV087 positively regulates innate immune response by promoting oligomerization of MITA/STING

**DOI:** 10.1128/jvi.01026-25

**Published:** 2025-10-09

**Authors:** Zhen-Zhen Li, Yu-Lin Yang, Meng-Yao Sun, Hong-Bing Shu, Li-Bo Cao

**Affiliations:** 1 College of Veterinary Medicine, Gansu Province Research Center for Basic Disciplines of Pathogen Biology; State Key Laboratory for Animal Disease Control and Prevention, Lanzhou Veterinary Research Institute, Chinese Academy of Agricultural Sciences, Lanzhou University 12426, , Lanzhou, China; 2 Department of Infectious Diseases, Medical Research Institute, Taikang Center for Life and Medical Sciences, Wuhan University, Zhongnan Hospital of Wuhan University 89674, Wuhan, China; Northwestern University Feinberg School of Medicine, Chicago, Illinois, USA

**Keywords:** lumpy skin disease virus, LSDV087, cGAS, MITA/STING, innate immunity

## Abstract

Lumpy skin disease (LSD), caused by the lumpy skin disease virus (LSDV), is a contagious disease in cattle that poses a major threat to the global cattle industry. The functions of most LSDV-encoded proteins remain poorly characterized, particularly regarding their roles in regulating innate immunity. In this study, we show that the LSDV-encoded protein LSDV087 positively regulates innate immune response independently of its decapping enzymatic activity. LSDV087 interacts with the adaptor protein MITA (also called STING) in the innate immune pathway, inhibits its degradation by reducing K48-linked polyubiquitination, and promotes its oligomerization and subsequent activation of downstream signaling events, leading to enhanced innate immune response. Consistently, LSDV087-deficient virus (LSDV∆087) exhibits an attenuated ability to activate cGAS-MITA-mediated innate immune response. Collectively, our study reveals regulatory mechanisms of LSDV-triggered innate immune response and points to the possibility of targeting LSDV087 for rational design of live-attenuated LSDV vaccines.

**IMPORTANCE:**

Lumpy skin disease virus (LSDV), which causes a contagious disease in cattle, poses a significant threat to the global cattle industry. Despite its impact, the functions of most LSDV-encoded proteins remain poorly understood. In this study, we report that LSDV087 plays dual roles in both promoting the cGAS-MITA-mediated innate immune response and downregulating host gene transcription. LSDV087 interacts with the adaptor protein MITA in the innate immune pathway, inhibits its degradation by reducing K48-linked polyubiquitination, and promotes its oligomerization, leading to the subsequent activation of downstream signaling events and an enhanced innate immune response. Additionally, as an immediate-early protein, LSDV087 functions as a decapping enzyme, preferentially targeting host transcripts with multiple exons to facilitate viral replication. This dual functionality underscores the complex interplay between LSDV immune evasion strategies and host defense mechanisms and may inform the rational design of live-attenuated LSDV vaccines.

## INTRODUCTION

Lumpy skin disease (LSD) is an acute, subacute, or chronic infectious disease caused by lumpy skin disease virus (LSDV), which mainly affects cattle and water buffaloes (1). Upon infection with LSDV, diseased cattle may experience reduced milk production, hide damage, abortions, temporary or permanent infertility, and even death, causing huge economic losses to the cattle farming industry (2, 3). LSD has been listed by the World Organization for Animal Health as a notifiable poxviral disease of significant impact that crosses national borders due to its widespread prevalence around the world (4, 5). Current studies on LSDV mainly focus on its epidemiology, etiology, diagnostic methods, and isolation (6–8). The LSDV genome is approximately 150 kilobases in length and encodes 156 predicted open reading frames (9). The functions of most LSDV-encoded proteins and the mechanisms by which LSDV interacts with the host immune system remain poorly understood, which limits the development of therapeutic drugs and effective vaccines for LSD. Innate immunity is crucial for the initial monitoring of invading viruses and primes subsequent adaptive immunity, which influences the intensity and quality of long-term protective immune responses against pathogens (10). Therefore, investigating the mechanisms of interaction between LSDV and the host innate immunity would provide potential strategies for the prevention and control of LSD.

The host innate immune system senses the invasion of pathogens via pattern recognition receptors (11–13). As a double-stranded DNA virus replicating in the cytoplasm of host cells, it is expected that LSDV genomic DNA is sensed by the cytosolic DNA sensor cGAS, a ubiquitously expressed cytoplasmic sensor for DNA (14–17). Upon sensing viral DNA, cGAS utilizes ATP and GTP to synthesize the second messenger cyclic GMP-AMP (cGAMP), which then binds to the endoplasmic reticulum (ER) associated adaptor protein MITA (also known as STING). This causes the conformational changes, oligomerization, and cellular trafficking of MITA. In this process, MITA recruits the downstream kinase TBK1 and transcription factor IRF3, leading to activation of IRF3, induction of type I interferons (IFNs), and other antiviral effector genes (18–21).

As a central adaptor protein in innate antiviral signaling, MITA is tightly regulated by post-translational modifications, including phosphorylation, sumoylation, and ubiquitination (22). Among them, ubiquitination plays a versatile role in regulating MITA activity (23). K63-linked polyubiquitination of MITA facilitates its oligomerization and ER-to-Golgi trafficking, enhancing downstream signaling and antiviral gene expression. Additionally, K63-linked polyubiquitination of MITA is implicated in its lysosomal degradation, serving as a feedback mechanism to avoid excessive immune activation (24, 25). Conversely, K48-linked polyubiquitination of MITA targets it for proteasomal degradation, thereby limiting its signaling activity and dampening the innate immune response (26–28).

Given the critical roles of the cGAS-MITA pathway in antiviral defense, identifying LSDV-encoded proteins that modulate this signaling axis is of great importance. In this study, we identified that LSDV-encoded LSDV087 protein positively regulated cGAS-MITA-mediated innate immune response independently of its decapping enzymatic activity. Mechanistically, LSDV087 interacted with MITA, reduced its K48-linked polyubiquitination and proteasomal degradation, and enhanced its oligomerization and signaling activity. Furthermore, deletion of LSDV087 attenuated LSDV-triggered innate immune response. Our findings reveal regulatory mechanisms of LSDV-triggered innate immune response, which may help in the rational design of live-attenuated LSDV vaccines.

## RESULTS

### LSDV087 positively regulates cGAS-MITA-mediated innate immune response

**Fig 1:**
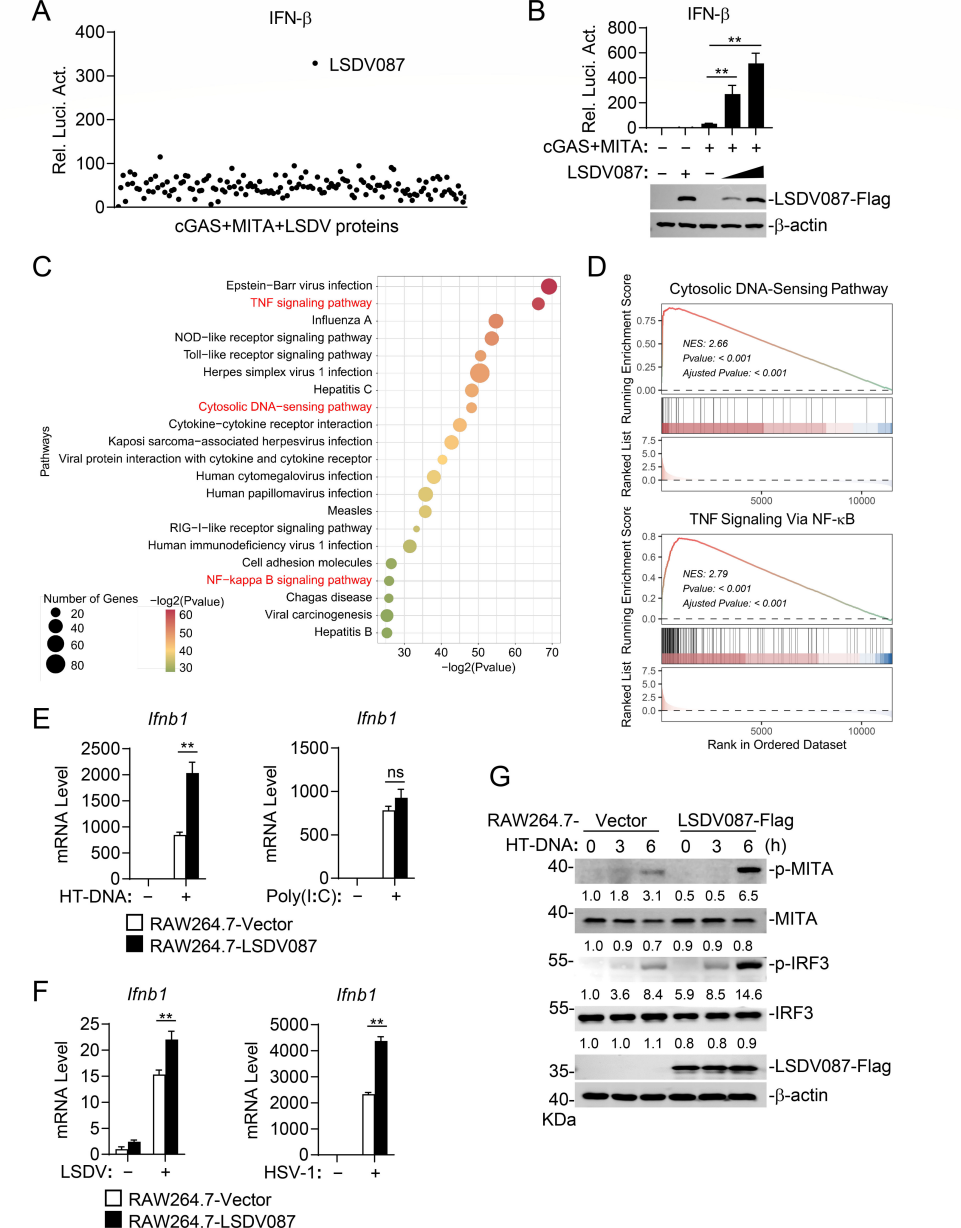
LSDV087 positively regulates cGAS-MITA-mediated innate immune response. (**A**) Screening of LSDV proteins that regulate activation of the IFN-β promoter induced by cGAS-MITA. HEK293 cells (1 × 10^5^) were transfected with pRL-TK (20 ng), IFN-β reporter (50 ng), cGAS (50 ng), MITA (10 ng), and LSDV expression plasmids (100 ng) for 24 hours before reporter assays. (**B**) Effects of LSDV087 on cGAS-MITA-induced activation of the IFN-β promoter. HEK293 cells (1 × 10^5^) were transfected with pRL-TK (20 ng), IFN-β reporter (50 ng), cGAS (50 ng), MITA (10 ng), and LSDV087 plasmid (50 and 100 ng) for 24 hours before reporter assays and immunoblot analysis with the indicated antibodies. (**C and D**) RNA-seq analysis of LSDV087-expressing and control RAW264.7 cells treated with HT-DNA. Kyoto Encyclopedia of Genes and Genomes analysis (**C**) and gene set enrichment analysis (GSEA) (**D**) of RNA-seq data were performed to identify biological signaling pathways enriched in RAW264.7 cells expressing LSDV087 compared to control cells treated with HT-DNA. (**E**) Effects of LSDV087 on HT-DNA- or poly(I:C)-triggered transcription of *Ifnb1* gene. LSDV087-expressing and control RAW264.7 cells (2 × 10^5^) were transfected with HT-DNA (2 µg) or poly(I:C) (2 µg) by lipofectamine for 6 hours before reverse transcription-quantitative PCR (RT-qPCR) analysis for mRNA levels of the *Ifnb1* gene. (**F**) Effects of LSDV087 on LSDV- or herpes simplex virus 1 (HSV-1)-triggered transcription of *Ifnb1* gene. LSDV087-expressing and control RAW264.7 cells (2 × 10^5^) were infected with LSDV for 9 hours or HSV-1 for 6 hours before RT-qPCR analysis for mRNA levels of the *Ifnb1* gene. (**G**) Effects of LSDV087 on HT-DNA-induced phosphorylation of MITA and IRF3. LSDV087-expressing and control RAW264.7 cells (2 × 10^5^) were transfected with HT-DNA for the indicated times before immunoblot analysis with the indicated antibodies. Band intensities were quantified by densitometry using ImageJ software and normalized to β-actin levels. Data shown in panels B, E, and F are mean ± SD (*n* = 3) from one representative experiment. All the experiments were repeated at least two times with similar results. ns, not significant; **P  <  0.01 (unpaired *t*-test).

During the long-term coevolution between LSDV and its hosts, the virus may evolve effector proteins that suppress the host innate immune response to promote viral replication. Conversely, some viral proteins may inadvertently activate host immunity, either as an unintended consequence or as part of a dynamic balance between host immune activation and viral immune evasion (29, 30). To identify LSDV proteins that regulate innate immune response, we constructed mammalian expression clones for 156 LSDV-encoded proteins and assessed their effects on activation of the IFN-β promoter induced by cGAS-MITA in reporter assays. This screen identified LSDV087 as a viral protein that enhances cGAS-MITA-induced IFN-β promoter activation (Fig. 1A). Further experiments indicated that overexpression of LSDV087 promoted cGAS-MITA-induced activation of the IFN-β promoter in a dose-dependent manner in HEK293 cells (Fig. 1B).

To investigate whether LSDV087 broadly regulates the expression of antiviral genes, we established a RAW264.7 cell line stably expressing LSDV087 through lentiviral transduction. Upon transfection with herring testis DNA (HT-DNA), RNA-seq analysis was performed to compare the transcriptional profiles between LSDV087-expressing and control cells. Differentially expressed genes were further analyzed through the Kyoto Encyclopedia of Genes and Genomes to explore their functions. Compared to the control cell line, 21 pathways were enriched in the LSDV087-expressing cell line, including the Cytosolic DNA-Sensing Pathway and the TNF Signaling Via NF-κB (TNF-NF-κB) pathways (Fig. 1C). Additionally, gene set enrichment analysis revealed that both the DNA-sensing and TNF-NF-κB pathways were upregulated in the LSDV087-expressing cell line compared to the control (Fig. 1D). These results suggest that LSDV087 broadly enhances DNA-induced transcription of antiviral genes. RT-qPCR analysis demonstrated that ectopically expressed LSDV087 promoted HT-DNA but not the double-stranded RNA analog poly(I:C)-triggered transcriptional induction of *Ifnb1* gene in RAW264.7 cells (Fig. 1E). Consistently, LSDV087 also promoted LSDV and another DNA virus herpes simplex virus 1 (HSV-1)-induced transcription of *Ifnb1* gene in RAW264.7 cells (Fig. 1F). Given that phosphorylation of MITA and IRF3 is critical in innate immune response to DNA, we further examined the effects of LSDV087 on these events. Immunoblot analysis indicated that LSDV087 promoted phosphorylation of MITA and IRF3 in response to HT-DNA (Fig. 1G). These results suggest that LSDV087 promotes LSDV-triggered innate immune signaling in human and mouse cell lines.

### LSDV087 deficiency attenuates LSDV-triggered innate immune response

**Fig 2:**
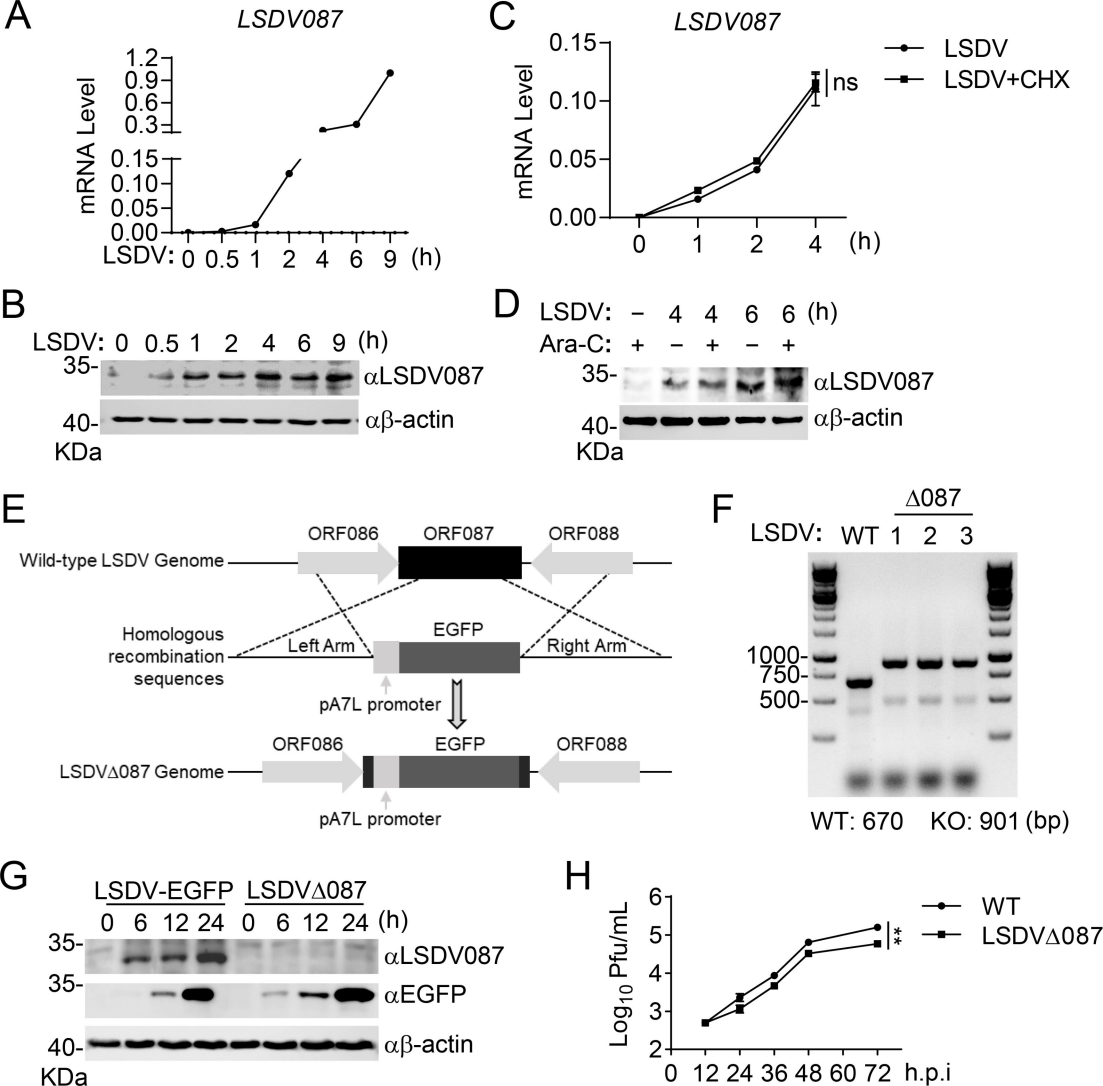
Construction and characterization of the LSDVΔ087 mutant strain. (**A**) Kinetics of *LSDV087* gene transcription. MDBK cells (3 × 10^5^) were infected with LSDV (multiplicity of infection [MOI] = 4) for the indicated times before RT-qPCR analysis of mRNA levels of the *LSDV087* gene. (**B**) Kinetics of LSDV087 protein expression. MDBK cells (3 × 10^5^) were infected with LSDV (MOI = 4) for the indicated times before immunoblot analysis with the indicated antibodies. (**C**) The effects of CHX on transcription of *LSDV087*. MDBK cells (3 × 10^5^) were infected with LSDV (MOI = 1) for the indicated times in the absence or presence of CHX (400 µM) before RT-qPCR analysis of mRNA levels of the *LSDV087* gene. (**D**) The effects of arabinoside C (Ara-C) on the LSDV087 protein level. MDBK cells (3 × 10^5^) were infected with LSDV (MOI = 1) for the indicated times in the absence or presence of arabinoside C (850 µM) before immunoblot analysis with the indicated antibodies. (**E**) Schematic diagram of the generation of LSDV strains with deletion of the *LSDV087* gene (LSDVΔ087). (**F**) PCR verification of LSDVΔ087 purity. Viral DNA of wild-type LSDV (WT) or LSDVΔ087 (MOI = 2) was amplified by PCR with a pair of primers specific for the LSDV genome before gel electrophoresis. (**G**) Immunoblot analysis of LSDV087 expression in wild-type and mutant LSDV-infected cells. MDBK cells (3 × 10^5^) were left uninfected or infected with wild-type LSDV-EGFP and LSDVΔ087 (MOI = 4) for the indicated times and then the expression of LSDV087 was detected by immunoblot analysis. (**H**) Growth kinetics of wild-type LSDV and LSDVΔ087. MDBK cells (1 × 10^5^) were infected with wild-type LSDV (WT) and LSDVΔ087 (MOI = 0.01) for the indicated times before plaque assays. Data shown in C and H are mean ± SD (*n* = 3) from one representative experiment, which was repeated at least two times with similar results. ns, not significant; **P  <  0.01 (unpaired *t*-test).

Given that overexpression of LSDV087 promotes LSDV-triggered innate immune response, we next investigated the effects of LSDV087 deficiency on LSDV-triggered innate immune response. RT-qPCR and immunoblot analysis indicated that LSDV087 mRNA and protein were detected at 0.5 hour post-infection (h.p.i.) of LSDV (Fig. 2A and B). The protein synthesis inhibitor cycloheximide (CHX) did not affect the early transcription of LSDV087 (Fig. 2C), and the DNA synthesis inhibitor arabinoside C (Ara-C) had no marked effect on its protein level at the early phase of infection (Fig. 2D). These results suggest that LSDV087 is an immediate-early gene of LSDV.

To investigate the roles of LSDV087 in the regulation of innate antiviral response, we generated a recombinant LSDV strain with deletion of the LSDV087 gene (LSDVΔ087) derived from the highly pathogenic LSDV/China/Hainan/2021 strain by homologous recombination (Fig. 2E). The purity of LSDVΔ087 was confirmed by PCR analysis of the viral genome (Fig. 2F), and the deficiency of LSDV087 in LSDV was verified by immunoblot analysis of LSDV087 level in cells infected with EGFP-tagged wild-type LSDV and LSDVΔ087 (Fig. 2G). The replication rates of LSDV∆087 were approximately twofold lower than those of wild-type LSDV at 36 and 48 h.p.i (Fig. 2H), suggesting a relatively weak effect of LSDV087 on LSDV replication, which may be attributed to its decapping activity.

**Fig 3:**
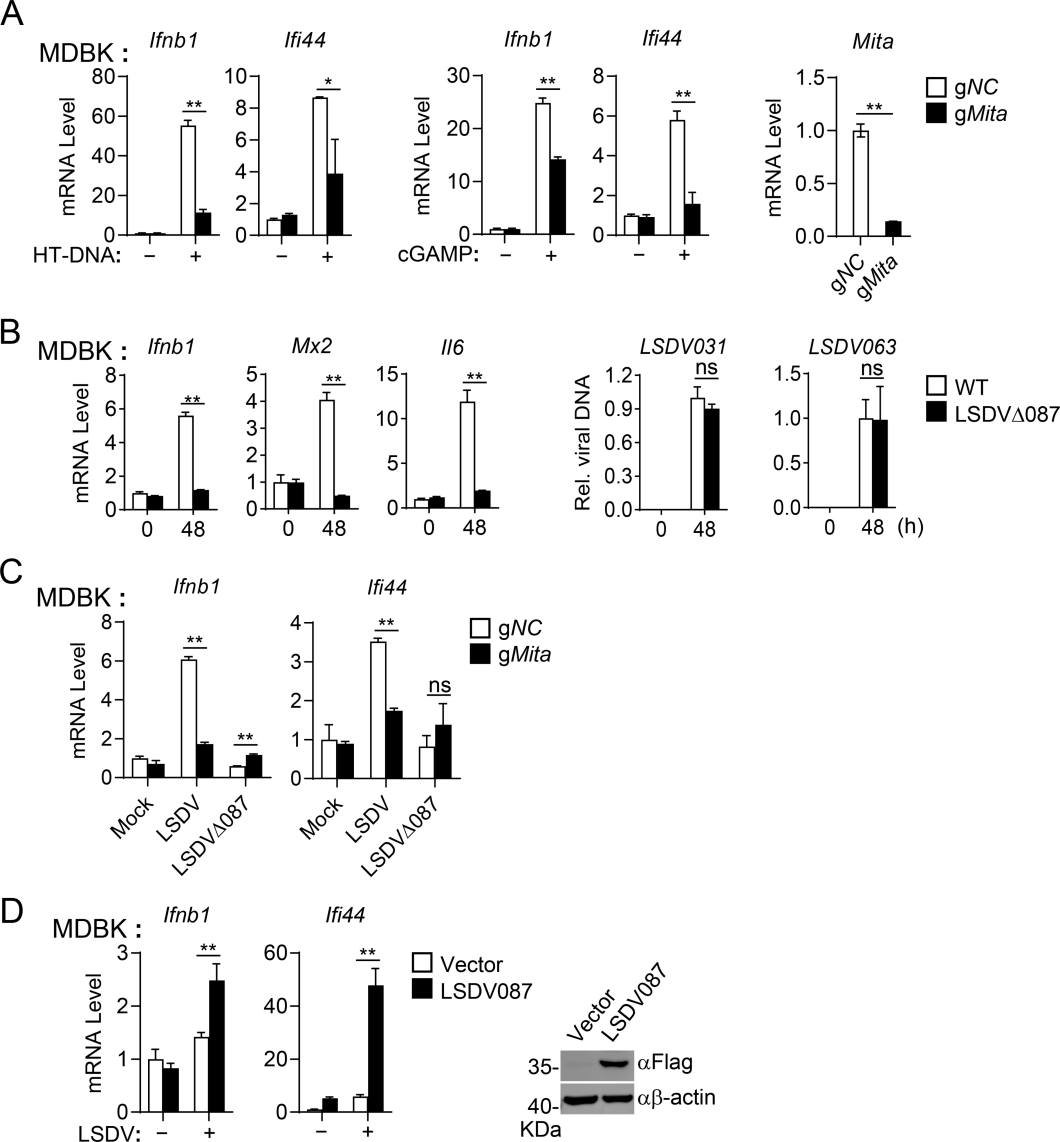
LSDV087 promotes LSDV-triggered cGAS-MITA signaling in MDBK cells (**A**) Effects of MITA deficiency on HT-DNA- or cGAMP-induced transcription of *Ifnb1* and *Ifi44* genes in MDBK cells. MITA-deficient (g*Mita*) and control (g*NC*) MDBK cells (2 × 10^5^) were mock-transfected or transfected with HT-DNA (2 µg) for 6 hours and were untreated or treated with cGAMP (0.3 µg) for 2 hours before RT-qPCR analysis of mRNA levels of the indicated genes. The knockout efficiency of MITA was assessed by RT-qPCR as indicated. (**B**) Effects of LSDV087 deficiency on LSDV-induced transcription of ISGs and viral replication. MDBK cells (2 × 10^5^) were left uninfected or infected with wild-type LSDV and LSDVΔ087 (MOI = 4) for 48 hours, followed by RT-qPCR analysis of mRNA levels of the indicated genes or qPCR analysis of *LSDV031* and *LSDV063* DNA levels. (**C**) Effects of MITA deficiency on LSDV- and LSDV087-induced transcription of ISGs. MITA-deficient (g*Mita*) and control (g*NC*) MDBK cells (2 × 10^5^) were left uninfected or infected with wild-type LSDV or LSDVΔ087 (MOI = 4) for 36 hours, followed by RT-qPCR analysis of mRNA levels of *Ifnb1* and *Iif44* genes. (**D**) Effects of LSDV087 on LSDV-induced transcription of downstream genes in MDBK cells. LSDV087-expressing and control MDBK cells (2 × 10^5^) were infected with LSDV for 36 hours before RT-qPCR analysis of mRNA levels of *Ifnb1* and *Iif44* genes and immunoblot analysis with the indicated antibodies. Data shown are mean ± SD (*n* = 3) from one representative experiment. All the experiments were repeated at least two times with similar results. ns, not significant; *P  <  0.05; **P  <  0.01 (unpaired *t*-test).

To investigate whether LSDV087 promotes DNA-triggered innate immune signaling in the natural host cells of LSDV, we performed experiments with Madin-Darby bovine kidney (MDBK) cells that have been shown to be infected by LSDV (31). In these cells, stimulation with HT-DNA or cGAMP induced transcription of *Ifnb1* and *Ifi44* genes, while knockout of MITA impaired it in MDBK cells (Fig. 3A). These results suggest that the cGAS-MITA pathway is functional in MDBK cells. Consistently, wild-type LSDV but not LSDVD087 induced transcription of interferon-stimulated genes (ISGs), including *Ifnb1*, *Mx2*, and *Il6* in MDBK cells at 48 h.p.i. In these experiments, the replication of LSDV structural genes *LSDV031* and *LSDV063* was not affected in MDBK cells at 48 h.p.i (Fig. 3B), suggesting that LSDV087 plays an important role in promoting LSDV-triggered induction of ISGs. Additionally, wild-type LSDV induced transcription of *Ifnb1* and *Ifi44* genes in control but not MITA-deficient MDBK cells, whereas LSDVΔ087 failed to induce transcription of these ISGs in both control and MITA-deficient MDBK cells (Fig. 3C). Conversely, in an MDBK cell line stably expressing LSDV087, LSDV-induced transcription of *Ifnb1 and Ifi44* genes was markedly enhanced compared to that of control MDBK cells (Fig. 3D). Together, these results suggest that LSDV087 enhances MITA-dependent antiviral innate immune response in bovine cells.

### LSDV087 regulates the cGAS-MITA signaling pathway independently of its decapping enzymatic activity

**Fig 4:**
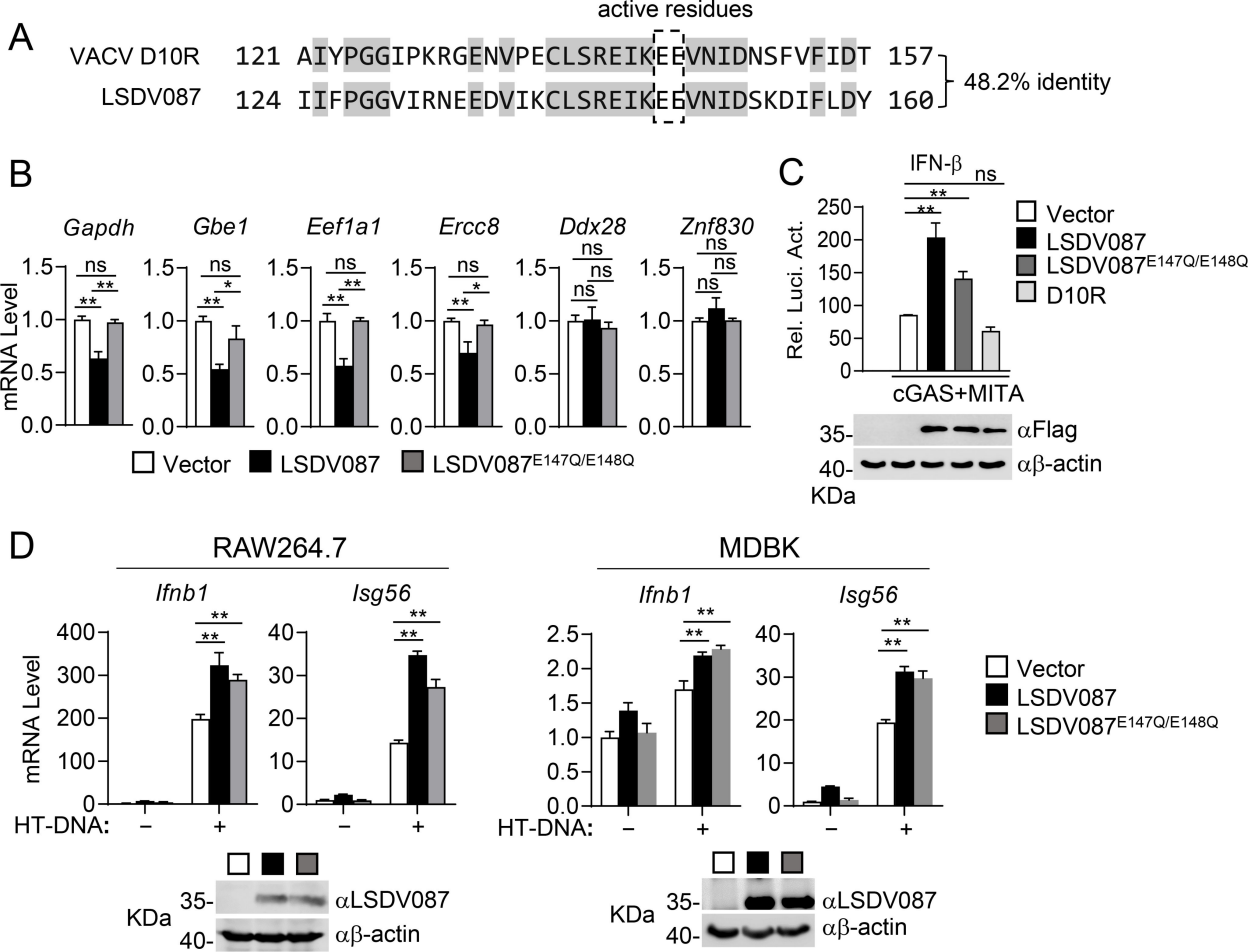
LSDV087 regulates cGAS-MITA signaling independently of its decapping enzymatic activity. (**A**) Partial sequence alignments of LSDV087 and VACV D10R. The conserved decapping enzyme active residues are circled. (**B**) Effects of LSDV087 and LSDV087^E147Q/E148Q^ on the mRNA levels of single- and multi-exonic genes. HEK293 cells (6 × 10^5^) were transfected with either an empty vector, LSDV087, or LSDV087^E147Q/E148Q^ plasmid for 36 hours before RT-qPCR experiments. (**C**) Effects of LSDV087, LSDV087^E147Q/E148Q^, and D10R on cGAS-MITA-induced activation of the IFN-β promoter. HEK293 cells (1 × 10^5^) were transfected with pRL-TK (20 ng), IFN-β reporter (50 ng), cGAS (50 ng), MITA (10 ng), and the indicated plasmids (100 ng) for 24 hours before reporter assays and immunoblot analysis with the indicated antibodies. (**D**) Effects of LSDV087 and LSDV087^E147Q/E148Q^ on HT-DNA-induced transcription of downstream genes in RAW264.7 and MDBK cells. Control, LSDV087- and LSDV087^E147Q/E148Q^-expressing RAW264.7 or MDBK cells were transfected with HT-DNA (2 µg) for 3 hours before RT-qPCR experiments and immunoblot analysis with the indicated antibodies. Data shown in panels B–D are mean ± SD (*n* = 3) from one representative experiment. All the experiments were repeated at least two times with similar results. ns, not significant; *P  <  0.05; **P  <  0.01 (unpaired *t*-test).

LSDV087 is annotated in the NCBI database as a homolog of the vaccinia virus (VACV) D10R, which downregulates the mRNA levels of multi-exonic genes in host cells through its decapping enzymatic activity (32, 33). Sequence alignment analysis suggests that LSDV087 shares 48.2% amino acid sequence identity with D10R and contains conserved decapping enzyme active residues at E147 and E148 (Fig. 4A). To investigate whether LSDV087 possesses decapping enzymatic activity similar to that of D10R, we examined the effects of LSDV087 and its E147Q/E148Q mutant (LSDV087^E147Q/E148Q^) on the mRNA levels of multi-exonic genes in HEK293 cells. RT-qPCR analysis indicated that overexpression of LSDV087 reduced the mRNA levels of multi-exonic genes, such as *Gapdh*, *Ercc8*, *Gbe1*, and *Eef1a1*, but barely affected the transcription of single-exon genes such as *Ddx28* and *Znf830*. In these experiments, LSDV087^E147Q/E148Q^ had no marked effects on mRNA levels of either single- or multi-exonic genes (Fig. 4B). These results suggest that LSDV087 functions similarly to D10R, selectively downregulating the transcription of multi-exonic genes.

To investigate whether the functions of LSDV087 in regulating antiviral innate immune response are dependent on its decapping enzymatic activity, we examined the effects of LSDV087^E147Q/E148Q^ on cGAS-MITA-triggered signaling. In reporter assays, both wild-type LSDV087 and LSDV087^E147Q/E148Q^, but not D10R, promoted activation of the IFN-β promoter induced by cGAS-MITA (Fig. 4C). We established RAW264.7 and MDBK cell lines stably expressing LSDV087 or LSDV087^E147Q/E148Q^ through lentiviral transduction. RT-qPCR experiments indicated that both wild-type LSDV087 and LSDV087^E147Q/E148Q^ enhanced HT-DNA-induced transcription of *Ifnb1* and *Isg56* genes in both RAW264.7 and MDBK cells (Fig. 4D). These results suggest that LSDV087 promotes cGAS-MITA signaling through a decapping activity-independent mechanism.

### LSDV087 positively regulates innate antiviral response by interacting with MITA

**Fig 5:**
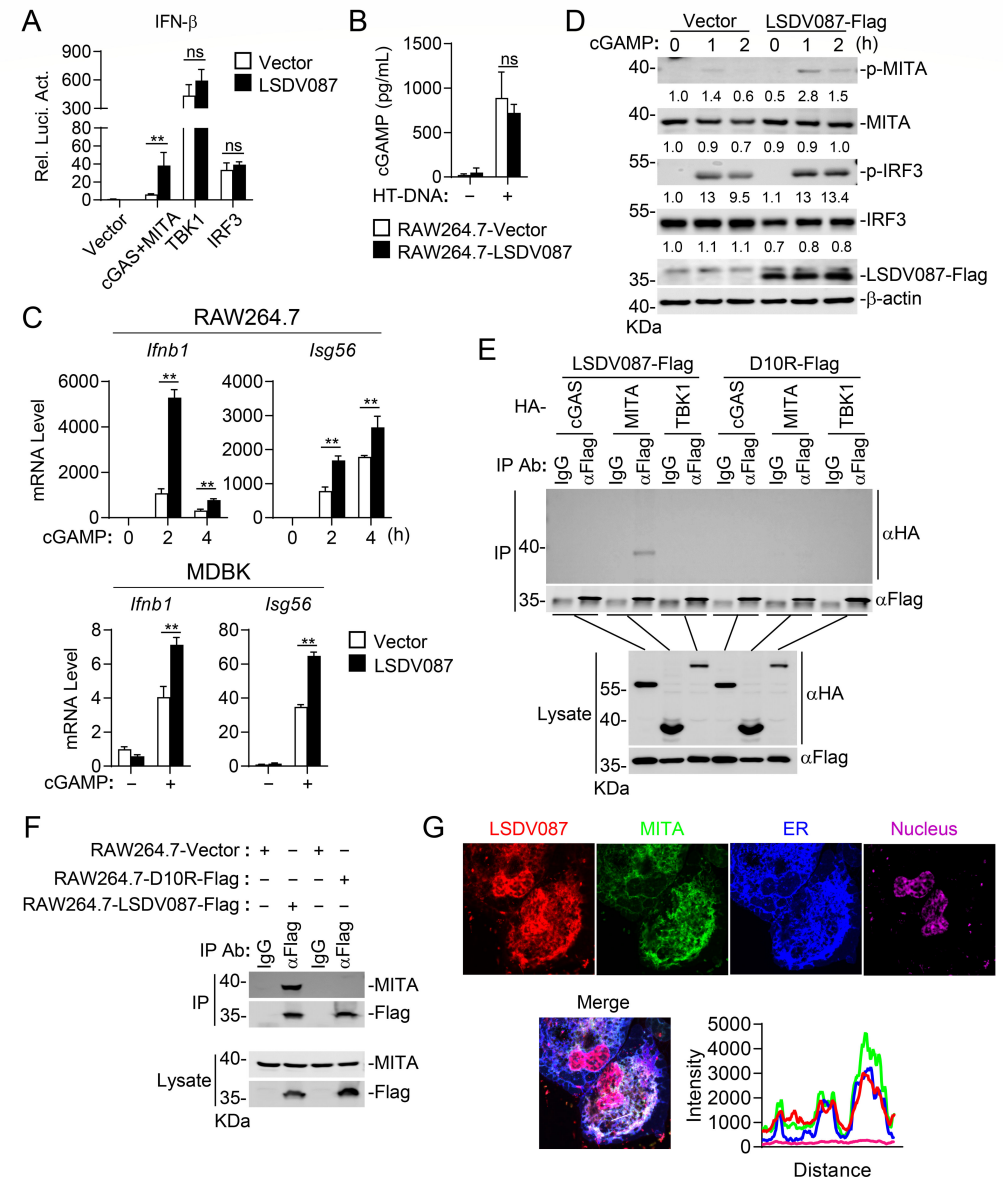
LSDV087 positively regulates innate antiviral response by interacting with MITA. (**A**) Effects of LSDV087 on activation of the IFN-β promoter mediated by various components. HEK293 cells (1 × 10^5^) were transfected with pRL-TK (20 ng), IFN-β reporter (50 ng), and the indicated plasmids for 24 hours before reporter assays. (**B**) Effects of LSDV087 on HT-DNA-induced cGAMP synthesis. LSDV087-expressing and control RAW264.7 cells (1 × 10^7^) were transfected with HT-DNA (2 µg) for 6 hours. cGAMP produced in the cells was analyzed using a cGAMP enzyme-linked immunosorbent assay (ELISA) kit. (**C**) Effects of LSDV087 on cGAMP-induced transcription of downstream genes. LSDV087-expressing and control RAW264.7 and MDBK cells were treated with cGAMP (0.3 µg) for the indicated times before RT-qPCR experiments. (**D**) Effects of LSDV087 on cGAMP-induced phosphorylation of MITA and IRF3. LSDV087-expressing and control RAW264.7 cells were treated with cGAMP (0.3 µg) for the indicated times before immunoblot analysis. Band intensities were quantified by densitometry using ImageJ software and normalized to β-actin levels. (**E**) Interaction of LSDV087 and D10R with various components in a mammalian overexpression system. HEK293 cells (1 × 10^7^) were transfected with the indicated plasmids for 24 hours and then lysed for co-immunoprecipitation with control IgG or anti-Flag, followed by immunoblot analysis with the indicated antibodies. (**F**) Interaction of LSDV087 and D10R with endogenous MITA. LSDV087- or D10R-expressing RAW264.7 cells (3 × 10^7^) were lysed for co-immunoprecipitation with control IgG or anti-Flag, followed by immunoblot analysis with the indicated antibodies. (**G**) Co-localization of LSDV087 with MITA. HeLa cells (2 × 10^4^) were transfected with the indicated plasmids for 20 hours and then fixed for immunostaining before being subjected to confocal microscopy. Data shown in panels A–C are mean ± SD (*n* = 3) from one representative experiment. All the experiments were repeated at least two times with similar results. ns, not significant; **P  <  0.01 (unpaired *t*-test).

To elucidate the mechanisms by which LSDV087 regulates the cGAS-MITA axis, we examined the effects of LSDV087 on activation of the IFN-β promoter mediated by overexpression of various components in the cGAS-MITA signaling pathway in reporter assays. The results indicated that LSDV087 promoted activation of the IFN-β promoter mediated by overexpression of cGAS and MITA but not by TBK1 or IRF3 (Fig. 5A). These results suggest that LSDV087 functions at the level of cGAS or MITA. We next investigated whether LSDV087 affects cGAS activity by measuring cGAMP production following HT-DNA stimulation. cGAMP synthesis assays showed that LSDV087 had no marked effects on HT-DNA-induced cGAMP synthesis (Fig. 5B), indicating that LSDV087 did not affect cGAS activity. However, LSDV087 promoted cGAMP-induced transcription of *Ifnb1* and *Isg56* genes in RAW264.7 and MDBK cells (Fig. 5C). Consistently, immunoblot analysis indicated that stable expression of LSDV087 enhanced cGAMP-induced phosphorylation of MITA and IRF3 in RAW264.7 cells (Fig. 5D). These results suggest that LSDV087 promotes antiviral innate immune response by modulating MITA activity.

Co-immunoprecipitation experiments indicated that LSDV087 interacted with MITA but not cGAS or TBK1, while D10R did not interact with any of them in a mammalian overexpression system (Fig. 5E). Furthermore, stably expressed LSDV087 but not D10R constitutively interacted with endogenous MITA in RAW264.7 cells (Fig. 5F). Confocal microscopy showed that the majority of LSDV087 colocalized with MITA in the ER under resting conditions (Fig. 5G). Collectively, these results suggest that LSDV087 regulates the cGAS-MITA axis by interacting with MITA.

### LSDV087 promotes MITA oligomerization

**Fig 6:**
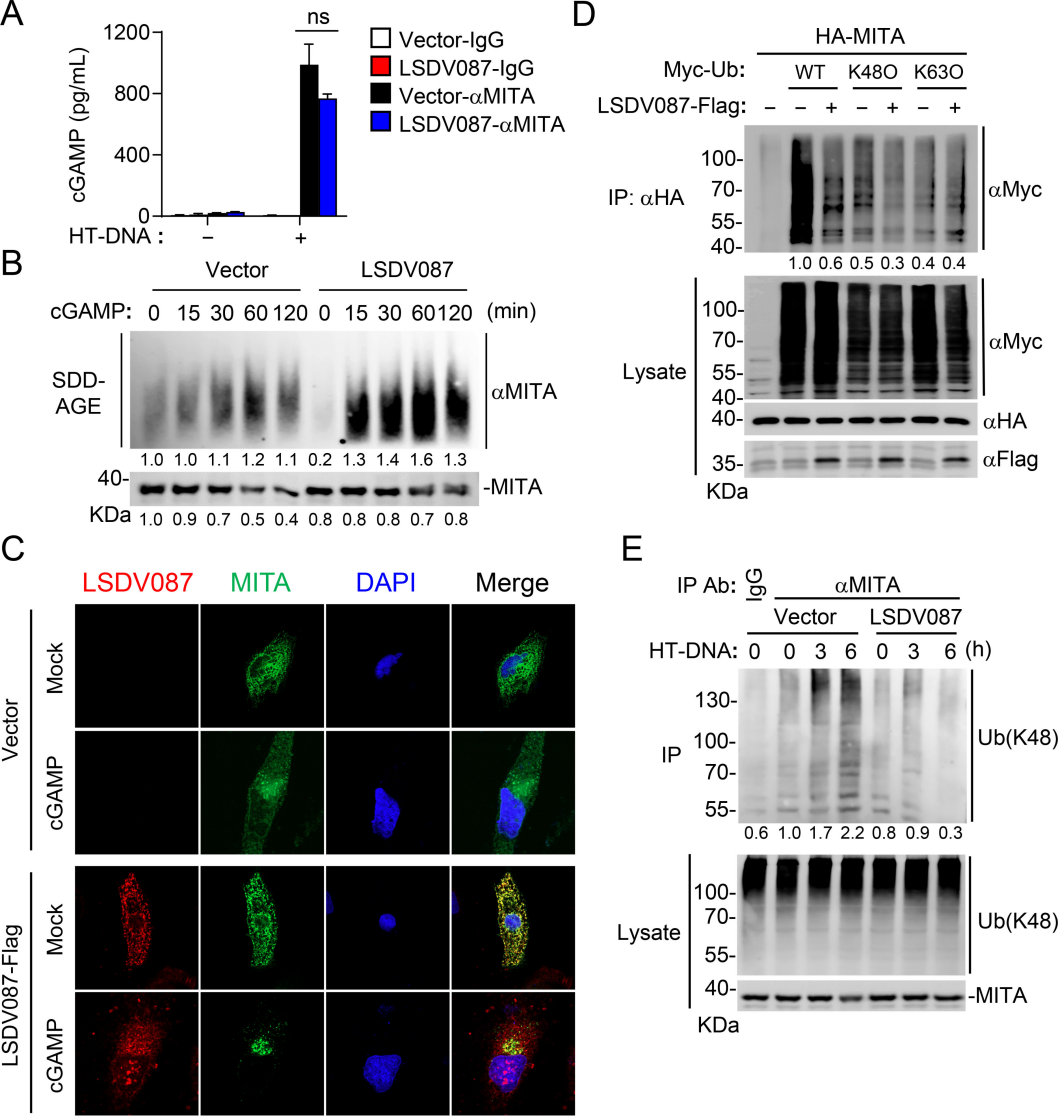
LSDV087 promotes MITA oligomerization. (**A**) Effects of LSDV087 on the binding of cGAMP to MITA. LSDV087-expressing and control RAW264.7 cells (3 × 10^7^) were transfected with HT-DNA (2 µg) for 6 hours and then lysed for co-immunoprecipitation with the indicated antibodies. cGAMP bound to immunoprecipitated MITA was measured by ELISA. Data shown are mean ± SD (*n* = 3) from one representative experiment. ns, not significant (unpaired *t*-test). (**B**) Effects of LSDV087 on cGAMP-induced MITA oligomerization. LSDV087-expressing and control RAW264.7 cells (3 × 10^7^) were treated with cGAMP (0.3 µg) for the indicated times before SDD-AGE experiments. Band intensities were quantified by densitometry using ImageJ software. (**C**) Effects of LSDV087 on cGAMP-induced aggregation of MITA. LSDV087-expressing and control HeLa cells (2 × 10^4^) were left untreated or treated with cGAMP (0.3 µg) for 30 minutes and then fixed for immunostaining before being subjected to confocal microscopy. (**D**) Effects of LSDV087 on K48-linked and K63-linked polyubiquitination of MITA. HEK293 cells (1 × 10^7^) were transfected with the indicated plasmids for 24 hours and then lysed for co-immunoprecipitation with control IgG or HA antibody, followed by immunoblot analysis with the indicated antibodies. Band intensities were quantified by densitometry using ImageJ software. (**E**) Effects of LSDV087 on HT-DNA-induced K48-linked polyubiquitination of endogenous MITA. LSDV087-expressing and control RAW264.7 cells (3 × 10^7^) were transfected with HT-DNA (2 µg) for the indicated times and then lysed for co-immunoprecipitation and immunoblot analysis with the indicated antibodies. All the experiments were repeated at least two times with similar results. Band intensities were quantified by densitometry using ImageJ software.

To investigate the molecular mechanisms by which LSDV087 regulates the activity of MITA, we examined the effects of LSDV087 on the binding of cGAMP to MITA. To do this, RAW264.7 cells stably transduced with an empty vector or LSDV087 were transfected with HT-DNA for 6 hours, and then cell lysates were immunoprecipitated with anti-MITA antibody. cGAMP bound to immunoprecipitated MITA was measured by ELISA. The results indicated that LSDV087 had no marked effects on the amounts of MITA-bound cGAMP (Fig. 6A). We next treated the LSDV087-expressing and control RAW264.7 cells with cGAMP and then examined the induction of MITA oligomerization by semi-denaturing detergent agarose gel electrophoresis (SDD-AGE) assays. The results indicated that LSDV087 promoted cGAMP-induced MITA oligomerization, which is an early step for its activation of downstream signaling events (Fig. 6B). In agreement with these biochemical results, confocal microscopy demonstrated that LSDV087 enhanced cGAMP-induced aggregation of MITA to form perinuclear punctate structures, which is a hallmark of activation of MITA-mediated downstream signaling (Fig. 6C). These results suggest that LSDV087 does not affect cGAMP binding to MITA but rather promotes MITA oligomerization and trafficking to form perinuclear punctate structures.

In our earlier experiments, we noticed that ectopic expression of LSDV087 inhibited the downregulation of MITA induced by HT-DNA or cGAMP (Fig. 1G, 5D and 6B). It has been reported that MITA undergoes K48-linked polyubiquitination for proteasomal degradation or K63-linked polyubiquitination for lysosomal degradation (24–28). Therefore, we next investigated the effects of LSDV087 on polyubiquitination of MITA. The results indicated that LSDV087 inhibited K48-linked but not K63-linked polyubiquitination of MITA in a mammalian overexpression system (Fig. 6D). Endogenous ubiquitination assays confirmed that LSDV087 inhibited HT-DNA-induced K48-linked polyubiquitination and downregulation of MITA (Fig. 6E). These results suggest that LSDV087 inhibits DNA-induced K48-linked polyubiquitination and degradation of MITA, which may contribute to its oligomerization and activation of downstream signaling.

## DISCUSSION

LSD is a significant transboundary disease that causes substantial economic losses to the livestock industry (34). Currently, there are no safe and effective commercial vaccines available for LSD. Although the attenuated vaccines derived from goat poxvirus and sheep poxvirus show some effectiveness in the prevention and control of LSD, their intradermal inoculation method requires a relatively high level of technical expertise from the operators, and there is also a potential risk of recombination with wild-type strains (35, 36). Therefore, elucidating the mechanisms by which LSDV interacts with the host immune system may help to develop new strategies to fight against LSD.

LSDV encodes 156 proteins, most of whose functions remain unclear. Through an unbiased screen by luciferase reporter assays, we discovered that LSDV087 promoted cGAS-MITA-mediated activation of the IFN-β promoter in HEK293 cells. Further studies revealed that overexpression of LSDV087 enhanced phosphorylation of MITA and IRF3 and transcription of downstream antiviral genes in response to HT-DNA and LSDV in mouse macrophage RAW264.7 cells. Overexpression of LSDV087 also increased LSDV-induced transcription of downstream antiviral genes in bovine MDBK cells. These findings suggest that LSDV087 positively regulates the cGAS-MITA signaling pathway.

RT-qPCR and immunoblot analysis indicated that LSDV087 is expressed at the early phase of LSDV infection. The protein synthesis inhibitor cycloheximide and the DNA synthesis inhibitor arabinoside C had no marked effects on its mRNA and protein levels at the early phase of infection. These results suggest that LSDV087 is an immediate-early gene of LSDV. To confirm the regulatory effects of LSDV087 on the innate immune response, we generated LSDVΔ087, an LSDV strain with deletion of the LSDV087 gene. Experimental results showed that deletion of LSDV087 had minimal (approximately twofold) effects on the production of progeny viruses and had no marked effects on the replication of viral DNA at 48 h.p.i. However, deficiency of LSDV087 fully impaired LSDV-induced transcription of downstream antiviral genes in bovine MDBK cells. These findings suggest that LSDV087 plays a positive regulatory role in LSDV-induced innate immune response. Poxviruses are well known for evolving various strategies to evade host innate immune response. However, our findings suggest that viral proteins do not always act solely as immune antagonists. During long-term host-pathogen coevolution, while viruses evolve immune evasion strategies, hosts may, in turn, evolve the ability to recognize and respond to essential viral proteins as part of their defense strategy.

LSDV087 is predicted to be a homolog of VACV D10R, and both proteins share conserved active residues of a decapping enzyme. Our experiments indicated that LSDV087 possessed decapping enzymatic activity similar to that of D10R. However, our experiments indicated that LSDV087 promoted the activation of the cGAS-MITA signaling pathway independently of its decapping enzymatic activity. Additionally, our results indicated that D10R did not interact with MITA and lacked the ability to positively regulate MITA-mediated signaling. These results suggest that the ability of LSDV087 in promoting innate antiviral response is not shared by homologs encoded by other poxviruses.

MITA is a key adaptor in DNA-induced innate immune response. Our results indicated that LSDV087 did not affect the synthesis of cGAMP or its binding to MITA upon DNA stimulation. However, LSDV087 interacted with MITA and enhanced cGAMP-induced oligomerization of MITA. LSDV087 inhibited DNA-triggered K48-linked polyubiquitination and degradation of MITA, which may contribute to its effects on MITA oligomerization and activation. Regrettably, due to the lack of a high-quality antibody against bovine MITA, we were limited in further biochemical experiments in LSDV-infected bovine cells.

Nevertheless, our study supports a model in which LSDV087 plays dual roles during LSDV infection. As an early viral protein, LSDV087 functions as a decapping enzyme, preferentially targeting host transcripts with multiple exons to facilitate viral replication. Additionally, LSDV087 interacts with MITA to promote its oligomerization and activation, leading to amplification of the host’s innate antiviral response. This dual functionality highlights the complex interplay between LSDV immune evasion strategies and host defense mechanisms.

## MATERIALS AND METHODS

### Reagents, cells, and viruses

Fetal bovine serum (SA211.02, Cellmax), penicillin and streptomycin (SV30010, HyClone), puromycin (AMR-J593,VWR), protein A + G agarose (P2055, Beyotime), PMSF (P7626, Sigma), Dual-Specific Luciferase Assay Kit (E1980, Promega), SYBR Green supermix (Q312, Vazyme), HiScript II Select RT SuperMix for qPCR (R323, Vazyme), isopropyl β-D-1-thiogalactopyranoside (IPTG) (ST098, Beyotime), HT-DNA (D6898, Sigma), Poly(I:C) (tlrl-pic, Invivogen), cGAMP ELISA Kit (CAY-501700-96S, Cayman), cGAMP (tlrl-nacga23-02, Invivogen), ATP (9804S, Cell Signaling Technology), GTP (51120, Sigma), digitonin (T2721, TargetMol), and aprotin, leupeptin, β-glycerophosphate disodium salt, Ara-C, CHX, and sodium orthovanadate (HY-P0017, HY-18234A, HY-126304, HY-13605, HY-12320, and HY-D0852, respectively, MCE) were purchased from the indicated manufacturers.

Mouse monoclonal antibody against HA (66006, Proteintech); rabbit monoclonal antibody against HA (H6908, Sigma); mouse monoclonal antibody against Flag (F3165, Sigma) and HRP-Flag (ZB15939, Servicebio); IgG (I5381, Sigma); antibodies against p-MITA (S366; 50907S, Cell Signaling Technology), MITA (A21051, ABclonal), p-IRF3 (S396; 4947S, Cell Signaling Technology), IRF3 (A19717, ABclonal), β-actin (A2228, Sigma-Aldrich); anti-mouse IgG (H + L), F(ab')2 Fragment (Alexa Fluor® 594 Conjugate; 8890, Cell Signaling Technology); Alexa Fluor 488 goat anti-rabbit IgG (H + L; A11008, Invitrogen); and 4′,6-diamidino-2-phenylindole (DAPI) (C1002, Beyotime) were purchased from the indicated manufacturers.

HEK293, RAW264.7, and HeLa cells were obtained from ATCC. MDBK cells were purchased from CCTCC. BEF cells, LSDV/China/Hainan/2021 strain, and EGFP-tagged wild-type LSDV were gifted by Mr. Huaijie Jia of Lanzhou Veterinary Research Institute. HSV-1 was previously described (37).

### Plasmids

The LSDV protein expression clones were synthesized by GenScript. Expression plasmids for Flag-tagged LSDV087, LSDV087^E147Q/E148Q^, LSDV087 (60–150), and VACV D10R were constructed by standard molecular biology techniques. Expression plasmids for HA-tagged cGAS, MITA, TBK1, and IRF3 were previously described (38).

### Generation of stable cell lines

A cDNA corresponding to LSDV087-coding sequence was cloned into the pCDH vector, which was co-transfected with packaging plasmids psPAX2 and pCMV-VSV-G into HEK293 cells. Thirty-six hours after transfection, the viruses were harvested for infection of RAW264.7 or MDBK cells. The infected cells were selected with puromycin (4 µg/mL) for 6 days to establish stable cell lines.

### Gene knockout by the CRISPR-Cas9 methods

Double-stranded oligonucleotides targeting *Mita* sequence were inserted into the lenti-CRISPR-V2 vector, which was co-transfected with packaging plasmids psPAX2 and pCMV-VSV-G into HEK293 cells. Thirty-six hours after transfection, the viruses were harvested for infection of MDBK cells. The infected cells were selected with puromycin (4 µg/mL) for 6 days to establish MITA-deficient cell lines. The following sequences were targeted for the bovine *Mita* gene.

5′-ACAGGCACTTAGCAGGACCA-3′.

### Generation of LSDVΔ087

LSDVΔ087 was generated by homologous recombination in MDBK cells. The recombinant plasmid (pUC18-LSDV087L-EGFP-LSDV087R) was first constructed using pUC18 used as the backbone. The cassette contains the left and right homology arms of *LSDV087* gene and the fluorescent gene *EGFP* under the control of the LSDV pA7L promoter, which was inserted into the middle of the reading frame of *LSDV087* gene. MDBK cells were infected with wild-type LSDV for 12 hours and then transfected with the homologous recombinant plasmid pUC18-LSDV087L-EGFP-LSDV087R using Lipo3000 transfection reagent. After culturing for 3 days, the cells were frozen-thawed and then seeded with MDBK cells in 96-well plates by limiting dilution. After several rounds of dilution screening and amplification, the purified LSDVΔ087 was obtained. The purity of LSDVΔ087 was determined by PCR and immunoblot analysis. The sequences of the PCR primers were as follows:

5′-CAAACGGAATAGAAAGTTGTCAAAAACATA-3′ and

5′-ACCCTTATTTCCAAAACATTTTAATCTTGA-3′.

### RT-qPCR and qPCR

Total RNAs and DNA were isolated for RT-qPCR or qPCR analysis to measure mRNA or viral DNA levels of the indicated genes, respectively. Data shown were the relative abundance of the indicated mRNA normalized to that of *Gapdh*. The sequences of the qPCR primers were as follows:

Human *Gapdh*: 5′-CAACGGATTTGGTCGTATTGG-3′ and

5′-GCAACAATATCCACTTTACCAGAGTTAA-3′;

Human *Gbe1*: 5′-AGTCGCTGGCATTTTGGTTG-3′ and

5′-AAGCCCATGCGTAATGAGTC-3′;

Human *Eef1a1*: 5′-TGTCGTCATTGGACACGTAGA-3′ and

5′-ACGCTCAGCTTTCAGTTTATCC-3′;

Human *Ercc8*: 5′-AAGGCAGTTTGCGCTAATGC-3′ and

5′-TGAGGCAGGATTTGGTCATACC-3′;

Human *Ddx28*: 5′-TCGTGACAGAGCAGAAAGGAC-3′ and

5′-TCATCAAGGCTGGCATTTGC-3′;

Human *Znf830*: 5′-AAGAATTGCGGCGGTTAATGA-3′ and

5′-CCCAAACGGTTGTACTTCGC-3′;

Mouse *Gapdh*: 5′-ACGGCCGCATCTTCTTGTGCA-3′ and

5′-ACGGCCAAATCCGTTCACACC-3′;

Mouse *Ifnb1*: 5′-TCCTGCTGTGCTTCTCCACCACA-3′ and

5′-AAGTCCGCCCTGTAGGTGAGGTT-3′;

Mouse *Isg56*: 5′-ACAGCAACCATGGGAGAGAATGCTG-3′ and

5′-ACGTAGGCCAGGAGGTTGTGCAT-3′;

Bovine *Gapdh*: 5′-AGGTCGGAGTGAACGGATTC-3′ and

5′-ATGGCGACGATGTCCACTTT-3′;

Bovine *Ifnb1*: 5′-TCCAGCACATCTTCGGCATT-3′ and

5′-AGACGATTCATCTGCCAATAGAGT-3′;

Bovine *Mx2*: 5′-CTACCGCAACATTACGCAGC-3′ and

5′-TCAGATCTGGGACCTCAGGG-3′;

Bovine *Il-6*: 5′-ACAAGCGCCTTCACTCCATT-3′ and

5′-GCGCTTAATGAGAGCTTCGG-3′;

Bovine *Isg56*: 5′-TCACAGCAACCATGAGTTATAAAG-3′ and

5′-ATCTCCTCCAAGACCCTGTT-3′;

Bovine *Ifi44*: 5′-ACAGTCTGCCCATTGCTGAA-3′ and

5′-CCACCATCTCATGGGAGAGC-3′;

Bovine *Mita*: 5′-CCCAAAAGGCAGCCTTGGTC-3′ and

5′-CCAGACTGCAGATTCCCTTG-3′;

*LSDV031*: 5′-ACAGTTGAATGTGATGGCGA-3′ and

5′-TGGGGATGAAGCTCTTGCAG-3′;

*LSDV063*: 5′-TGTTCATTCACCATCCGCATC-3′ and

5′-GGTTCTTGTAATGGCTTGTTGC-3′;

*LSDV087*: 5′-AAACTGTCTTCGTCAGACCAT-3′ and

5′-CGTTTCTAATTACCCCACCTGGA-3′.

### Transfection and reporter assays

HEK293 cells were transfected with the indicated plasmids by the calcium phosphate precipitation method. To normalize for transfection efficiency, a pRL-TK (Renilla luciferase) reporter plasmid was added to each transfection. Luciferase assays were performed using a dual-specific luciferase assay kit. Firefly luciferase activities were normalized on the basis of Renilla luciferase activities.

### Fluorescent confocal microscopy

HeLa cells were seeded on coverslips in 24-well plates and transfected with the indicated plasmids for 24 hours. LSDV087-expressing and control RAW264.7 cells were seeded and treated with cGAMP for 30 minutes. The cells were fixed with 4% paraformaldehyde for 20 minutes and then permeabilized with 0.1% Triton X-100 for 15 minutes. Subsequently, the cells were blocked in 1% bovine serum albumin (BSA) and stained with the indicated antibodies. Fluorescence imaging was performed using a Zeiss confocal microscope.

### Co-immunoprecipitation and immunoblot analysis

Cells were lysed with lysis buffer (20 mM Tris-HCl pH 7.4, 1% NP-40, 150 mM NaCl, and 1 mM EDTA and protease inhibitors) at 4°C for 10 minutes and sonicated for 1 minute. The lysates were centrifuged at 13,000 rpm for 10 minutes at 4°C. The supernatants were immunoprecipitated with the indicated antibodies. Then the beads were washed three times with washing buffer (750 mM NaCl and 50 mM Tris-HCl pH 7.4). The bound proteins were separated by SDS-PAGE, followed by immunoblot analysis with the indicated antibodies.

### Semi-denaturing detergent agarose gel electrophoresis

RAW264.7 cells were lysed in NP-40 lysis buffer, and the cell lysates were mixed in sample buffer (0.5× Tris-borate-EDTA [TBE] buffer, 10% glycerol, 2% SDS, and 0.0025% bromophenol blue) and loaded onto a vertical 2% agarose gel (Bio-Rad). After electrophoresis in the running buffer (1× TBE and 0.1% SDS) for about 2 hours with a constant voltage of 100 V at 4°C, the proteins were transferred to an Immobilon membrane (Millipore) for immunoblot analysis.

### Preparation of LSDV087 antibody

The prokaryotic expression vector pET-30c-LSDV087 (60–150) was transformed into *E. coli* BL21 (DE3) competent cells. A 10 mL Luria-Bertani (LB) medium starter culture was inoculated from a single colony and grown overnight at 37°C with shaking at 220 rpm. The culture was then diluted 1:50 into 500 mL LB medium and incubated until reaching mid-log phase (OD_600_ = 0.6–0.8). Protein expression was induced with 0.5 mM IPTG at 16°C for 10 hours. The recombinant protein was purified under denaturing conditions following inclusion body isolation protocols. The purified protein was used as an antigen to immunize 8-week-old BALB/c mice via multi-site subcutaneous injections. Mice were immunized at 14-day intervals for a total of five doses. Polyclonal antibodies were harvested from serum samples collected 1 week after the final immunization.

### cGAMP treatment

cGAMP was delivered into RAW264.7 or MDBK cells using a digitonin-based permeabilization method. Briefly, cells were pretreated with digitonin permeabilization buffer (50 mM HEPES, pH 7.0; 100 mM KCl; 3 mM MgCl_2_; 0.1 mM dithiothreitol (DTT); 85 mM sucrose; 0.2% BSA; 1 mM ATP; 0.1 mM GTP; and 10 µg/mL digitonin) at 37°C for 20 min, followed by incubation with cGAMP.

### cGAMP activity assay

Raw264.7 cells were either untreated or transfected with HT-DNA for 6 hours. Cell lysates were then prepared and heated at 95°C for 10 minutes to denature proteins, followed by centrifugation at 20,000 g for 25 minutes at 4°C. The supernatants containing cGAMP were collected and analyzed using a cGAMP ELISA kit. The cGAMP level was measured as an indicator of cGAS activity.

### Viral plaque assay

MDBK cells were infected with wild-type LSDV and LSDVΔ087 (MOI = 0.01) for the indicated times. Both cells and supernatants were harvested and freeze-thawed to obtain viral suspensions. Serial dilutions of these suspensions were used to infect MDBK cells for 2 hours at 37°C. The cells were then overlaid with 1.5% methylcellulose and incubated for 96 hours before plaque counting.

### Facility biosafety statement

All experiments involving live LSDV were conducted in an ABSL-3 laboratory at the Lanzhou Veterinary Research Institute of the Chinese Academy of Agricultural. Environmental and equipment decontamination were performed after each experiment according to institutional biosafety protocols. The experiments were approved by the Ministry of Agriculture and Rural Affairs (approval number: 07140020250302) and the China National Accreditation Service for Conformity Assessment (approval number: CNAS BL0098). We acknowledge the ABSL-3 staff for their operational and logistical support.

### Statistics analysis

Unpaired Student’s *t*-test was used for statistical analysis with GraphPad Prism Software. The number of asterisks represents the degree of significance with respect to *P* values. Statistical significance was set at *P* < 0.05 (*) and *P* < 0.01 (**).

## Data Availability

The RNA-seq data in this study are available in the NCBI BioProject database under accession number PRJNA1286107.
